# The science of safety: complications associated with the use of mechanical circulatory support in cardiogenic shock and best practices to maximize safety

**DOI:** 10.12688/f1000research.25518.1

**Published:** 2020-07-29

**Authors:** Navin K. Kapur, Evan H. Whitehead, Katherine L. Thayer, Mohit Pahuja

**Affiliations:** 1 The Cardiovascular Center for Research and Innovation, Tufts Medical Center, Boston, MA, USA; 2 Division of Cardiology, Detroit Medical Center/Wayne State University School of Medicine, Detroit, MI, USA

**Keywords:** Safety review, cardiogenic shock, acute mechanical circulatory support, impella, ecmo, intraaortic balloon pump

## Abstract

Acute mechanical circulatory support (MCS) devices are widely used in cardiogenic shock (CS) despite a lack of high-quality clinical evidence to guide their use. Multiple devices exist across a spectrum from modest to complete support, and each is associated with unique risks. In this review, we summarize existing data on complications associated with the three most widely used acute MCS platforms: the intra-aortic balloon pump (IABP), Impella systems, and veno-arterial extracorporeal membrane oxygenation (VA-ECMO). We review evidence from available randomized trials and highlight challenges comparing complication rates from case series and comparative observational studies where a lack of granular data precludes appropriate matching of patients by CS severity. We further offer a series of best practices to help shock practitioners minimize the risk of MCS-associated complications and ensure the best possible outcomes for patients.

## Acute mechanical circulatory support in cardiogenic shock

Cardiogenic shock (CS) is an advanced state of hemodynamic compromise representing a convergent endpoint of cardiac decompensation resulting from acute myocardial infarction, end-stage heart failure, myocarditis, and various other conditions. In-hospital mortality in CS remains unacceptably high, with recent estimates ranging from 27–51% ^1^. Historically, CS has been managed largely with intravenous inotropes and vasopressors, medications which improve systemic perfusion at the cost of worsening myocardial supply/demand imbalance. Despite seeming to temporarily improve hemodynamic indices and traditional markers of tissue perfusion, empiric studies have shown that escalating inotropes and vasopressors fail to meaningfully reverse the downward hemodynamic spiral that occurs in CS, with very poor survival observed in patients requiring multiple agents ^2^.

Mechanical circulatory support (MCS) devices are commonly used to augment cardiac output and decouple systemic perfusion from native myocardial energy expenditure in CS. The intra-aortic balloon pump (IABP) was first applied in CS in the late 1960s, promising to improve hemodynamics through balloon counterpulsation synchronized with the cardiac cycle ^3^. The IABP became widely used and gained a class I indication for CS until the landmark IABP-SHOCK II trial showed that it failed to improve short- or long-term mortality in acute myocardial infarction complicated by CS (AMICS) ^4– 6^. Over the past decade, several other acute MCS platforms capable of providing much greater support have been developed and adopted to varying degrees in clinical practice. The trans-valvular axial flow pumps such as the Impella device (Abiomed, Danvers, MA) directly unload the left ventricle, capable of providing between 3 and 5.5 L/minute of flow while reducing native myocardial oxygen demand ^7^. A percutaneous right ventricular Impella device (Impella RP) is also available, which bypasses the right ventricle by displacing blood from the right atrium to pulmonary artery ^8^.

The TandemHeart device (CardiacAssist, Pittsburgh, PA) consists of a left atrial drainage cannula connected to an extracorporeal centrifugal flow pump, which returns blood to the descending aorta at flow rates of up to 5 L/minute ^7^. Because of the technical complexity and complications associated with the need for trans-septal puncture for delivery of the left atrial drainage cannula, the TandemHeart device is less commonly used in contemporary practice and thus will be only briefly discussed here. Finally, veno-arterial extracorporeal membrane oxygenation (VA-ECMO), a peripheral modification of cardiopulmonary bypass, is the only device which provides full systemic circulatory and respiratory support, though at the cost of increased left ventricular afterload ^9^. Recognizing the futility of escalating inotropes as well as the imperative to effectively intervene before impaired systemic perfusion progresses to an irreversible state of widespread metabolic failure, we and others have proposed CS management algorithms which incorporate early application of these advanced MCS platforms, with device selection tailored to the individual patient’s hemodynamic profile ^10– 13^.

## Putting recent safety signals into context

All MCS devices are associated with risk. This risk is increased among patients in CS who are commonly treated with vasoconstrictive agents, anticoagulants, and anti-platelet drugs, and may be exposed to other devices requiring large bore access such as hemodialysis catheters, pulmonary artery catheters, and other venous or arterial sheaths for monitoring and drug delivery. Use of large-bore MCS access along with a requirement for systemic anticoagulation increases the risk for bleeding, limb ischemia, and stroke ^14^. Intravascular shear and varying degrees of hemocompatibility with non-biologic surfaces can induce hemolysis, which in combination with systemic inflammation and thromboembolism can predispose to renal failure, with significant prognostic implications ^15^. Understanding the relative risks of these various complications with different MCS devices and their implications for device selection in patients has been extremely challenging.

Straightforward comparison of complication rates across devices is possible only by using data from randomized trials in which equivalent patients are randomized to different device strategies. Complication rates observed in randomized trials performed to date are summarized in Table 1. The IABP-SHOCK II trial was the largest randomized trial of an MCS device performed in CS to date and reported low rates of bleeding, limb ischemia, and stroke in both the IABP and medical therapy arms ^4^. Two small randomized trials comparing the TandemHeart device to IABP showed a clear signal toward higher rates of bleeding and limb ischemia with TandemHeart, though these trends were significant in only one of the two studies ^16, 17^. Three small, underpowered randomized trials have been conducted evaluating the use of Impella in CS. The ISAR-SHOCK (Efficacy Study of LV Assist Device to Treat Patients with Cardiogenic Shock) trial was a small study (n = 25) comparing the early generation Impella 2.5 to IABP, powered for a surrogate endpoint of hemodynamic improvement 30 minutes after device insertion ^18^. A numerically higher incidence of bleeding, hemolysis, and limb ischemia was observed among Impella patients, though the sample was too small to evaluate the significance of these trends. The IMPRESS in Severe Shock (IMPella versus IABP Reduces mortality in STEMI patients treated with primary PCI in Severe cardiogenic SHOCK) trial was designed to compare all-cause mortality between IABP and the Impella CP in AMICS but ended up being completed as an exploratory safety trial because of miscalculation of expected event rates ^19^. A trend toward higher rates of bleeding and hemolysis was observed with the Impella CP, though the small sample size limited any conclusive findings. Finally, the IMPELLA-STIC trial compared IABP alone to IABP plus Impella 5.0 in 12 patients with AMICS and found a significantly higher rate of major bleeding in the combined device group, though the study was too small to evaluate the significance of other complication trends ^20^. To date, only one randomized trial has evaluated the use of VA-ECMO in CS: the Extracorporeal Life Support in cardiogenic Shock complicating acute myocardial infarction (ECLS-SHOCK) trial randomized 42 post-arrest AMICS patients to VA-ECMO or no MCS and found similar rates of complications between groups, though again the study was underpowered for clinical outcomes including safety conclusions ^21^.

**Table 1.** Summary of MCS complication rates reported in randomized trials and comparative observational studies of acute MCS in CS. AKI, acute kidney injury; AMICS, acute myocardial infarction complicated by cardiogenic shock; CS, cardiogenic shock; CVA, cerebrovascular accident; GI, gastrointestinal; GU, genitourinary; HgB, hemoglobin; IABP, intra-aortic balloon pump; MCS, mechanical circulatory support; n.s., not significant; OR, odds ratio; PCI, percutaneous coronary intervention; pRBCs, packed red blood cells; RU, rectal ulcer; TIA, transient ischemic attack; VA-ECMO, veno-arterial extracorporeal membrane oxygenation.

Study	Comparison	Major bleeding	Stroke	AKI	Limb ischemia	Hemolysis	Sepsis	Death	Perspective
Thiele *et al*. (IABP-SHOCK- II) ^4^		*Life-threatening* *or severe and* *moderate* *bleeding as* *defined by* *GUSTO criteria*	*New* *neurologic* *symptoms in* *conjunction* *with signs of* *ischemia or* *hemorrhage* *on head CT*	*x*	*Peripheral* *ischemic vascular* *complication* *requiring surgical* *or interventional* *management*	*x*	*Sepsis with* *clinical signs* *of infection* *and elevated* *procalcitonin* *levels*	*30-day* *mortality*	• Poorly defined controls No definition of shock severity • Wide range of timing for IABP support initiation • No hemodynamic guidance for management of shock • 1-year and 6- year mortality also assessed and showed no difference
	IABP (n = 301)	Life-threatening: 3.3% Moderate: 17.3%	0.7% (2/300)	-	4.3% (13/300)	-	15.6% (47/300)	39.70%	
	Medical therapy (n = 299)	Life- threatening: 4.4% Moderate: 16.4%	1.7% (5/298)	-	3.4% (10/298)	-	20.5% (61/298)	41.30%	
	*P* value	0.51	0.28	-	0.53	-	0.15	0.69	
Thiele *et al*. ^16^		*Major bleeding* *requiring* *transfusion* *of blood* *components*	*CVA with* *neurological* *dysfunction*	*x*	*Lower extremity* *ischemia requiring* *surgical or* *interventional action*	*x*	*Elevated body* *temperature* *>38.5°C*	*30-day* *mortality*	• Surrogate primary endpoint • Not sufficiently powered to assess mortality • AMICS only • MCS implantation was prior or post PCI
	IABP (n = 20)	40% (8/20)	Not reported	-	0% (0/20)	-	50% (10/20)	45%	
	Tandem (n = 21)	90.5% (19/21)	Not reported	-	33.3% (7/21)	-	81% (17/21)	43%	
	*P* value	0.002	-	-	0.009	-	0.08	0.86	
Burkhoff *et al*. ^17^		*Bleeding*	*Neurologica* *dysfunction*	*Renal* *dysfunction*	*Distal leg ischemia*	*Plasma free Hgb >40* *mg/dL two or more* *measurements taken* *8 hours apart*	*Systemic infection* *or sepsis*	*30-day* *mortality*	• Surrogate primary endpoint • Trial ended prematurely • Did not exclude patients with IABP already placed at time of enrollment
	IABP (n = 14)	14.3% (2/14)	50% (7/14)	21.4% (3/14)	14.3% (2/14)	7.1% (1/14)	35.7% (5/14)	64%	
	Tandem (n = 19)	42.1% (8/19)	31.6% (6/19)	21.5% (4/19)	21.5% (4/19)	5.3% (1/19)	21% (4/19)	53%	
	*P* value	0.13	0.47	0.99	0.99	0.99	0.44	n.s	
Seyfarth *et al*. (ISAR- SHOCK) ^18^		*Units of pRBCs* *administered* *per patient*	*Survival* *without* *neurologic* *deficit*	*x*	*Acute limb ischemia* *requiring surgery*	*Time course of free* *HgB*	*x*	*30-day* *mortality*	• Not powered fo mortality • AMICS only • Impella 2.5 does not provide complete support and may not be an appropriate comparison to IABP • Definition of major bleeding makes it difficult to compare to other studies • Safety outcomes were not well reported • Outcomes of patients who died prior to intervention were included in analysis
	IABP (n = 13)	1.2 U	30.8% (4/13)	-	0% (0/13)	-	-	46.20%	
	Impella 2.5 (n = 12)	2.6 U	50% (6/12)	-	8.3% (1/12)	-	-	50%	
	*P* value	0.18	Not reported	-	Not reported	-	-	Not reported	
Ouweneel *et al*. (IMPRESS) ^19^		*Serum Hgb* *drop of 5 g/dL* *or transfusion* *of two units* *of pRBCs* *or surgery* *to control* *bleeding*	*Any stroke* *confirmed by* *neurologist* *and CT scan*	*x*	*Major bleed at* *arterial access site* *requiring device* *extraction or* *thrombotic occlusion* *of femoral artery* *or limb ischemia* *requiring device* *extraction or need* *for vascular surgery* *to correct vascular* *complication*	*Evidence of clinically* *relevant hemolysis* *requiring device* *extraction*	*x*	*30-day* *mortality*	• Underpowered due to poor estimation of true mortality rate • Study cohort not appropriate for treatment evaluation; single MCS intervention alone is unlikely to benefit severe CS patients • No safety *p* values reported so difficult to assess comparison
	IABP (n = 24)	8.3% (2/24)	4.2% (1/24)	-	0% (0/24)	0% (0/24)	-	50%	
	Impella CP (n = 24)	33.3% (8/24)	4.2% (1/24)	-	4.2% (1/24)	33.3% (8/24)	-	45.80%	
	*P* value	Not reported	Not reported	-	Not reported	Not reported	-	0.92	
Bochaton *et al*. (IMPELLA- STIC) ^20^		*Major bleeding* *requiring* *transfusion*	*x*	*x*	*Limb complication*	*x*	*Sepsis*	*30-day* *mortality*	• Severely inadequate sample size due to slow enrollment • Compounding effect of having IABP in both arms • Vague definitions of safety outcomes
	IABP (n = 6)	0% (0/6)	-	-	0% (0/6)	-	50% (3/6)	0%	
	IABP + Impella 5.0 (n = 6)	83.3% (5/6)	-	-	33.3% (2/6)	-	83.3% (5/6)	33.30%	
	*P* value	0.02	-	-	0.46	-	0.59	0.46	
Schrage *et al*. ^24^		*Life-threatening* *or severe and* *moderate* *bleeding as* *defined by* *GUSTO criteria*	*New* *neurologic* *symptoms in* *conjunction* *with signs of* *ischemia or* *hemorrhage* *on head CT*	*x*	*Peripheral* *ischemic vascular* *complication* *requiring surgical* *or interventional* *management*	*x*	*Sepsis with* *clinical signs* *of infection* *and elevated* *procalcitonin* *levels*	*30-day* *mortality*	• Non-randomized study • Included Impella 2.5 and Impella CP • Only 38.1% of Impellas were implanted prior to PCI • Poorly defined control group
	No Impella (n = 237)	Life- threatening: 3.0% Moderate: 16.9%	2.5% (6/237)	-	3.8% (9/237)	-	19.4% (46/237)	46.40%	
	Impella 2.5/CP (n = 237)	Life- threatening: 8.4% Moderate: 20.3%	2.5% (6/237)	-	9.7% (23/237)	-	30.8% (73/237)	48.50%	
	*P* value	Life- threatening: <0.01 Moderate: 0.32	0.76	-	0.01	-	<0.01	0.64	
Amin *et al*. ^25^		*Bleeding* *event requiring* *transfusion*	*Ischemic* *stroke,* *hemorrhagic* *stroke,* *intracerebral* *hemorrhage,* *or TIA*	*AKI*	*x*	*x*	*x*	*In-hospital* *mortality*	• Observational study • Did not control for many parameters that may affect association with mortality • Only reported ORs, no absolute values to assess actual effect
	Impella (4,782) versus IABP (43,524), propensity matched	OR: 1.10 (1.0–1.21)	OR: 1.34 (1.18–1.53)	OR: 1.08 (1.0–1.17)	-	-	-	OR: 1.24 (1.13–1.36)	
Dhruva *et al*. ^26^		*Hgb drop <3 g/* *dL, transfusion* *of whole blood* *or pRBCs,* *procedural* *intervention to* *treat bleeding,* *or transfusion* *of whole blood* *or packed* *red blood, or* *suspected* *GI, GU, RP, or* *other bleed*	*x*	*x*	*x*	*x*	*x*	*In-hospital* *mortality*	• Observational study • Crude imputation of missing registry data • Patients with multiple PCIs included in study and not accounted for • Minimal safety outcome assessment • Did not control for many parameters that may affect association with mortality
	Impella (n = 1,680)	31.3% (526/1,680)	-	-	-	-	-	45.0%	
	IABP (n = 1680)	16.0% (268/1,680)	-	-	-	-	-	34.1%	
	*P* value	<0.001	-	-	-	-	-	<0.001	
	IABP (n = 7,805)	14.5%	-	-	-	-	-	28.6%	
	No device (n = 7,805)	11.0%	-	-	-	-	-	26.5%	
	*P* value	<0.001	-	-	-	-	-	0.002	
Brunner *et al*. (ECLS- SHOCK) ^21^		*Life-* *threatening,* *severe, or* *moderate* *bleeding*	*Stroke*	*x*	*Peripheral* *ischemic vascular* *complication*	*x*	*Sepsis*	*30-day* *mortality*	Study is not well described, particularly control group definition, and may not be an appropriate comparator
	No MCS (21)	14.3% (3/21)	4.8% (1/21)	-	0% (0/21)	-	33.3% (7/21)	19%	
	VA-ECMO (21)	19.0% (4/21)	4.8% (1/21)	-	9.5% (2/21)	-	42.9% (9/21)	33.30%	
	*P* value	1.0	1.0	-	0.49	-	0.75	0.37	

Given the paucity of sufficiently powered randomized trials, most data on complication rates come from observational case series and registries, which are summarized in Table 2. Despite inconsistent reporting and variability in outcome definitions, several trends are apparent from these data. First, higher rates of bleeding and vascular injury are associated with devices requiring larger bore access including Impella (13–14 French access sheaths) and VA-ECMO (21–27 French venous cannulas plus 15–21 French arterial cannulas) compared to IABP (8–9 French access). Similarly, rates of stroke and limb ischemia are increased with Impella compared with IABP, and higher still with VA-ECMO. Two recent analyses by Pahuja *et al.* comparing rates of stroke, bleeding, and limb ischemia among AMICS patients treated with IABP, percutaneous VADs (Impella or TandemHeart), or VA-ECMO are exemplary of these trends. In this large sample, stroke was observed in 3.1% of patients with IABP, 5.6% of those with pVADs, and 9.7% of those treated with VA-ECMO. Similarly, bleeding occurred in 19.4%, 29.9%, and 54.2% of each group, respectively, while limb ischemia occurred in 0.9%, 3.6%, and 7.7%, respectively. All of these complications were associated with increased length of stay and hospitalization costs ^22, 23^. While it stands to reason that more invasive devices would lead to higher complication rates, it must be noted that these observational studies do not account for significant baseline differences between the real-world patients in whom the devices are deployed. Crude insight about the severity of shock across device cohorts can be gained by comparing the short-term mortality rates in Table 2. The lowest mortality is observed in patients treated with IABP (24%), which may be related to the selection of patients with “less-severe” CS who do not require high levels of hemodynamic support. In contrast, higher flow devices such as Impella may be chosen for patients with more severe CS, as reflected in a significantly higher mortality rate of 43.1%. Finally, as it is the only device capable of providing both circulatory and respiratory support, VA-ECMO is often applied emergently for patients with cardiac arrest or severe CS, reflected in an extremely high observed mortality rate of 57.2%. Some of the observed disparity in complication rates across device cohorts may therefore be driven by baseline differences in illness severity, in addition to device factors. Accordingly, lower complication rates have been observed when these advanced devices have been applied in less-severely-ill cohorts. The recent STEMI-Door-to-Unload (STEMI-DTU) pilot trial tested the safety and feasibility of Impella CP use in 50 patients with anterior STEMI, without CS. In this cohort with 4% overall mortality, complication rates were quite low: bleeding occurred in 14%, stroke in 2%, renal dysfunction in 4%, and hemolysis in 2% ^89^. Two patients (4%) had major vascular events related to flow-limiting dissections of the femoral artery at device removal, with no device related mortality observed.

**Table 2.** Complication rates associated with IABP, Impella, and VA-ECMO from observational studies in cardiogenic shock. Death represents either 30-day or in-hospital mortality, whichever was reported in the individual study. AKI, acute kidney injury; IABP, intra-aortic balloon pump; VA-ECMO, veno-arterial extracorporeal membrane oxygenation

IABP	Study	n	Bleeding (%)	Stroke (%)	AKI (%)	Limb ischemia (%)	Hemolysis (%)	Sepsis (%)	Death (%)
	Tehrani *et al*. ^10^	55	9.1	-	23.6	7.3	0.0	-	-
	Alushi *et al*. ^33^	54	7.4	1.8	-	0.0	-	-	52.0
	Pieri *et al*. ^34^	36	36.1	8.3	-	2.8	0.0	36.0	6.0
	Ferguson *et al*. ^35^	16,909	2.4	-	-	2.9	-	-	21.2
	Cohen *et al*. ^36^	22,663	0.9	-	-	0.9	-	-	21.3
	Stone *et al*. ^37^	5,495	4.3	-	-	2.3	-	-	20.0
	Siriwardena *et al*. ^38^	645	2.9	2.3	16.6	2.6	-	-	-
	Cohen *et al*. ^39^	1,119	4.6	3.3	-	-	-	-	-
	Valente *et al*. ^40^	414	7.2	-	-	2.4	-	-	-
	Davidicius *et al*. ^41^	360	19.0	-	-	4.0	-	-	-
	Ternus *et al*. ^42^	682	0.6	-	-	1.3	0.0	-	18.5
	Schwartz *et al*. ^43^	50	24.0	4.0	-	6.0	-	-	34.0
	Mackenzie *et al*. ^44^	100	2.0	-	-	25.0	-	-	40.0
	Ozen *et al*. ^45^	3,135	1.4	-	-	12.3	0.7	-	25.9
	Arceo *et al*. ^46^	212	2.4	-	-	5.7	-	-	45.0
	Dick *et al*. ^47^	187	-	-	-	2.5	-	-	7.2
	Eltchaninoff *et al*. ^48^	240	3.3	-	-	12.9	-	0.4	24.0
	Meisel *et al*. ^49^	161	2.5	-	-	2.5	-	-	7.2
	Pahuja *et al*. ^22, 23^	86,796	19.4	3.1	-	0.9	-	-	25.8
	**Weighted average**		**12.9**	**3.1**	**17.2**	**1.5**	**0.6**	**5.0**	**24.2**
Impella		n	Bleeding (%)	Stroke (%)	AKI (%)	Limb ischemia (%)	Hemolysis (%)	Sepsis (%)	Death (%)
	Tehrani *et al*. ^11^	67	4.5	-	25.4	4.5	10.5	-	-
	Annamalai *et al*. ^50^	34	20.6	5.9	47.1	8.8	11.8	-	38.0
	Alushi *et al*. ^33^	62	14.5	1.6	-	8.0	-	-	67.0
	O’Neill *et al*. ^51^	154	20.1	1.9	-	-	-	-	-
	Jensen *et al*. ^52^	109	59.0	0.0	-	10.1	11.9	-	-
	Karatolios *et al*. ^53^	27	62.9	-	-	3.7	-	-	-
	Karami *et al*. ^54^	90	23.3	4.4	-	2.2	6.7	-	52.2
	Pieri *et al*. ^34^	28	35.7	7.1	-	18.0	32.0	29.0	21.0
	Basir *et al*. ^55^	171	7.0	-	-	4.1	-	28.0	-
	Ternus *et al*. ^42^	96	5.2	-	-	2.1	1.0	-	30.2
	Kaki *et al*. ^56^	17	5.9	5.9	41.0	5.9	-	-	70.6
	Lauten *et al*. ^57^	120	28.4	1.7	31.7	-	7.5	-	64.2
	Ouweneel *et al*. ^58^	112	25.0	3.6	-	-	7.1	-	65.0
	Badiye *et al*. ^59^	40	-	-	42.0	-	62.5	-	32.5
	Esposito *et al*. ^60^	23	-	-	-	-	30.4	-	57.0
	Schwartz *et al*. ^43^	7	57.0	0.0	-	0.0	-	-	14.0
	Garan *et al*. ^12^	31	-	12.9	-	12.9	-	-	45.2
	Lamarche *et al*. ^61^	29	-	-	-	0.0	-	-	38.0
	Anderson *et al*. ^62^ (RP)	60	48.3	-	-	-	21.7	-	26.7
	Pahuja *et al*. ^22^,23	2,079	29.9	5.6	-	3.6	-	-	41.0
	**Weighted average**		**27.7**	**4.9**	**34.1**	**4.2**	**13.1**	**28.1**	**43.1**
ECMO		n	Bleeding (%)	Stroke (%)	AKI (%)	Limb ischemia (%)	Hemolysis (%)	Sepsis (%)	Death (%)
	Tehrani *et al*. ^11^	31	16.1	-	51.6	19.4	38.7	-	-
	Karami *et al*. ^54^	38	31.6	10.5	-	5.3	0.0	-	47.4
	Chamogeorgakis *et al*. ^63^	61	-	-	-	13.1	-	-	50.8
	Hoefer *et al*. ^64^	131	11.5	2.3	-	1.5	-	-	-
	Koerner *et al*. ^65^	184	22.3	4.9	16.3	4.3	9.2	18.5	61.0
	Lorusso *et al*. ^66^	4,522	-	5.4	-	-	-	-	-
	Garan *et al*. ^12^	20	-	25.0	-	10.0	-	-	45.0
	Kolla *et al*. ^67^	27	16.0	7.4	56.0	-	-	-	70.0
	Gulkarov *et al*. ^68^	71	4.1	14.1	45.1	19.7	-	-	53.5
	Yau *et al*. ^69^	154	-	-	-	22.0	-	-	59.7
	Avalli *et al*. ^70^	100	-	-	-	35.0	-	-	72.0
	Wong *et al*. ^71^	193	18	9.0	-	11.0	-	-	61.0
	Ranney *et al*. ^72^	80	-	-	-	21.3	-	-	60.0
	Foley *et al*. ^73^	43	-	-	-	16.3	-	-	79.0
	Lamb *et al*. ^74^	91	-	-	-	13.2	-	-	58.0
	Belle *et al*. ^75^	51	39.2	3.9	-	17.6	13.7	13.7	72.5
	Bermudez *et al*. ^76^	42		11.9	40.5	14.3	-	-	62.0
	Kim *et al*. ^77^	27	14.2	-	37.0	-	-	-	41.7
	Esper *et al*. ^78^	18	94.4	5.6	-	22.2	-	-	33.0
	Loforte *et al*. ^79^	73	50.7	15.1	52.0	54.8	-	15.1	54.8
	Moraca *et al*. ^80^	26	-	7.7	34.6	7.7	-	-	35.0
	Pagani *et al*. ^81^	33	-	0.9	30.3	-	-	-	64.0
	Wu *et al*. ^82^	60	-	-	31.7	10.0	-	-	47.0
	Formica *et al*. ^83^	42	54.8	26.2	47.6	21.4	-	-	61.9
	Lamarche *et al*. ^61^	32	-	-	-	15.6	-	-	44.0
	Batra *et al*. ^84^	1,286	32.3	-	21.1	-	-	-	54.1
	Vallabhajosyula *et al*. ^85^	4,608	25.3	10.8	-	-	-	-	57.7
	Pahuja *et al*. ^22, 23^	444	54.2	9.7	-	7.7	-	-	55.9
	**Weighted average**		**28.2**	**8.2**	**25.6**	**14.3**	**11.8**	**16.9**	**57.2**

A final class of studies which have been used to compare relative complication rates between devices are comparative observational analyses, which attempt to adjust for the significant baseline differences observed in cases series by attempting to match patients across available clinical variables. Three such studies have recently spurred substantial debate about complications related to Impella use. Schrage *et al.* performed a matched analysis comparing 237 CS patients treated with Impella in 13 European centers to 237 matched patients taken from both arms (IABP and medical therapy) of the IABP-SHOCK II trial ^24^. Amin *et al.* identified patients undergoing PCI with MCS (linked by same-day billing data), some of whom had CS, and performed a propensity matched analysis comparing those managed with Impella with those managed by IABP ^25^. Finally, Dhruva *et al.* linked two registries to identify AMICS patients managed with Impella or IABP, matched for 75 baseline variables (though, notably, lactate and hemodynamic parameters were not available for matching) ^26^. Each study reported a higher rate of complications associated with Impella compared to IABP. Even more striking, the Amin and Dhruva studies reported a significantly higher rate of in-hospital mortality in patients treated with Impella, raising the concern that the additional complications associated with Impella may translate to higher mortality. While these analyses corroborate the trends observed in observational case series as summarized in Table 2, their salience ultimately depends on the validity of their respective mortality comparisons. As previously discussed, it is relatively obvious why larger sheath sizes would cause higher rates of bleeding and limb ischemia; whether morbidity from these complications outweighs the benefits afforded by greater hemodynamic support in patients with severe shock for whom an IABP would be inadequate remains unanswered. We argue that this question can be properly answered only through trials comparing CS patients randomized to different devices and powered for hard clinical endpoints. No amount of propensity matching—particularly when the most well-validated prognostic variables in CS such as central venous pressure, lactate, and cardiac power output are missing—can account for the vast baseline differences between real-world patients being managed with these different devices. The ongoing DanGer shock trial and other trials may shed light on these critical questions ^27^.

## Moving forward without data

In the absence of high-quality randomized data clarifying the net risks and benefits of MCS platforms in CS, practitioners still need to move forward and manage individual patients with CS. Recognizing that there are likely higher rates of at least some complications (bleeding, limb ischemia, and hemolysis) with Impella and VA-ECMO compared to IABP, shock practitioners should redouble their efforts to adhere to the following best practices.

### Rapid, coordinated multi-specialty evaluation of patients with suspected cardiogenic shock

Institutions should design structured responses (such as the ‘Shock Team’) to ensure that patients with suspected CS are rapidly identified and evaluated by qualified practitioners so that necessary resources can be urgently made available and evaluations begun regarding the likelihood of recovery or candidacy for durable MCS or transplant. Depending on the suspected inciting insult and local staffing patterns, the shock team might include interventional cardiologists, heart failure specialists, (cardiac) intensivists, and cardiac/vascular surgeons along with perfusionists and critical care nurses. Some have suggested that the cath lab be used as a default staging ground where right heart catheterization, coronary angiography, and fluoroscopic MCS insertion can all be rapidly performed ^11^.

### Comprehensive invasive hemodynamic assessment to guide device selection and management

**Figure 1.**
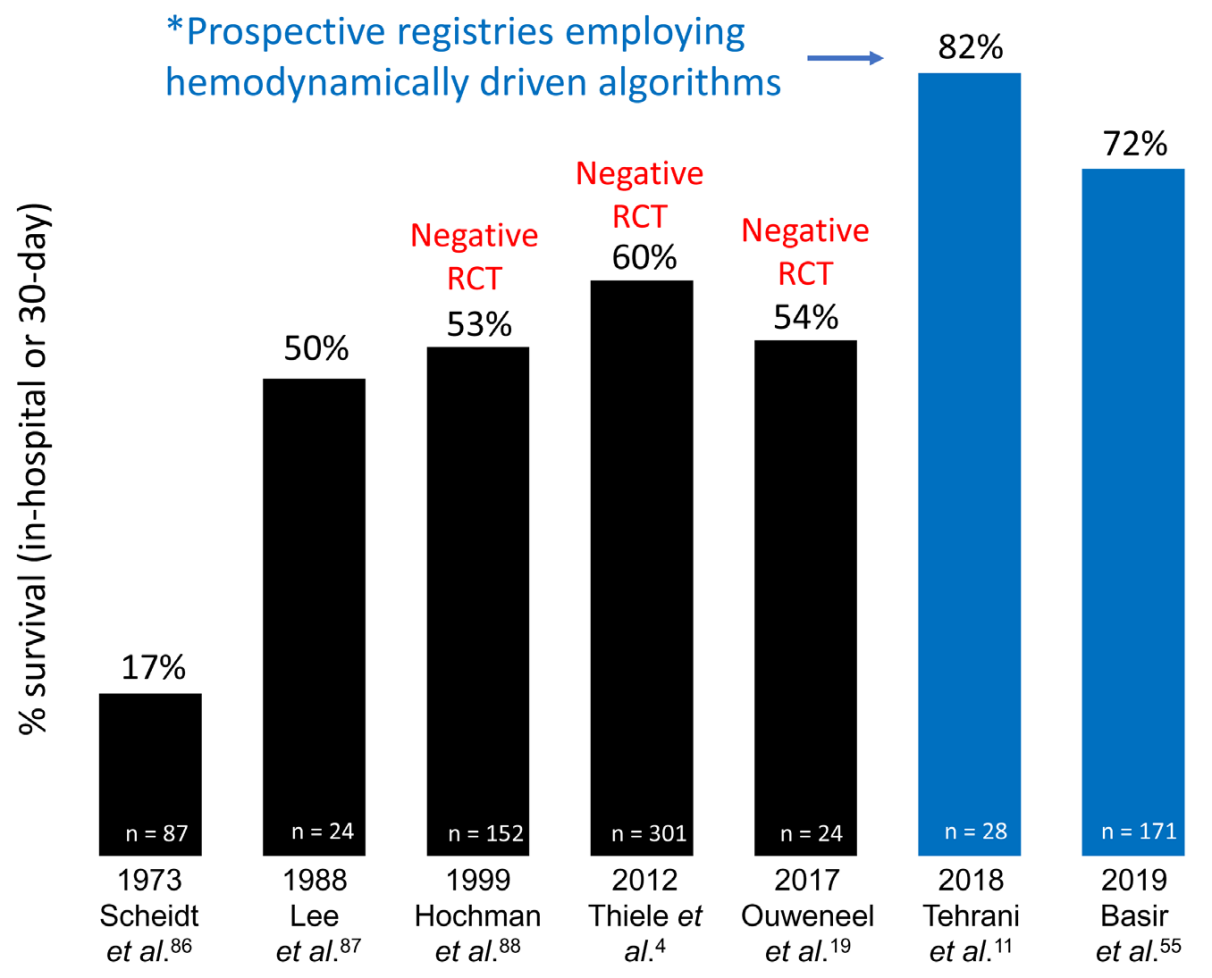
Survival and studies in acute myocardial infarction cardiogenic shock. Two recent prospective registries employing a hemodynamically driven treatment algorithm have reported higher survival rates compared to several recent randomized controlled trials (RCTs) that failed to use hemodynamic data to guide device selection or device management.

Critical to maximizing the risks and benefits of MCS is the use of objective hemodynamic assessment using pulmonary artery catheters (PACs) to guide device selection. Just as it is important to identify the crashing patient with severe biventricular congestion who is unlikely to stabilize with IABP alone, it is equally important to identify the STEMI patient with isolated left ventricular failure well-suited for left-sided Impella, sparing them the additional complications of a more invasive device like VA-ECMO. We and others have proposed algorithms for device selection based on the ventricular congestive profile and validated indices of right ventricular function such as pulmonary artery pulsatility index (PAPi), along with the presence of concurrent respiratory failure ^10, 12^. Recent data suggest that algorithms incorporating hemodynamically guided decision-making may lead to improved survival in AMICS ( Figure 1), though observational studies directly examining associations between PAC use and clinical outcomes in CS have yielded mixed results ^11, 28– 31^.

### Fastidious prevention of and monitoring for the development of complications

Many complications can be avoided or their impact minimized if recognized and managed promptly. For example, a careful vascular assessment should be performed daily to monitor for signs of limb ischemia, which may require intervention such as external bypass or addition of a distal perfusion catheter. Similarly, markers of hemolysis should be continually tracked to assess the need for device repositioning and thorough neurologic exams performed to identify signs of stroke, which can be particularly difficult to recognize in unconscious patients. In ECMO patients, right upper extremity oxygen saturation and pulmonary capillary wedge pressure should be continuously monitored for the development of Harlequin syndrome and left ventricular distension, which may require optimization of ventilator settings, upgrade to a VAV-ECMO configuration, or addition of a left ventricular vent ^9^. We would refer readers to a comprehensive review by Subramaniam and colleagues for further discussion of risk factors and strategies to reduce complications from acute MCS devices ^14^.

### Continuous re-assessment to guide device weaning or escalation

Aside from complications occurring at the time of device insertion or removal, most occur as a function of time on support. Multimodal data (labs, hemodynamic parameters, echocardiography) should be continuously re-integrated to assess for the possibility of device weaning or the need for escalation. Specific thresholds (cardiac power output <0.6, PAPi <0.9) have been proposed to guide consideration of escalation or addition of right-sided support, though specific device algorithms will depend on local availability ^30^.

### Implementation of best practices for device removal

Large bore access devices above 17Fr are commonly referred to vascular surgery for removal. However, with emerging techniques, devices ranging from 12Fr to 17Fr may be removed with percutaneous closure approaches. The Perclose device (Abbott Inc) can be used at the time of device implantation (pre-closure approach) or at the time of device removal (post-closure approach) to rapidly achieve hemostasis. The Manta closure device (Teleflex Inc) has recently been introduced and may represent another approach for percutaneous vascular hemostasis. The introduction of the Impella CP with a side-arm access port allows for device removal and post-closure, thereby mitigating vascular complication risk at the time of device removal ^32^. Each operator must develop these technical skills to improve outcomes.

## Conclusion

CS is a complex clinical syndrome that remains a major cause of global mortality and morbidity. MCS device utilization is growing for cardiogenic shock, though each MCS platform is associated with risks. High-quality randomized controlled trials evaluating the use of MCS devices for CS are currently lacking but are in development. Recent reports utilizing administrative datasets and retrospective registries are of limited value other than to raise awareness that randomized controlled trials are needed to improve outcomes for patients. Progress in the field will be made only when high-quality randomized trials are conducted in defined populations, powered for all-cause mortality. The decision to place an advanced device should be made by an experienced shock practitioner or team using the most complete information possible. In the meantime, clinicians should educate themselves about hemodynamically driven decision-making algorithms and best practices to reduce complications associated with each MCS platform.
